# Rapid appraisal of barriers to the diagnosis and management of patients with dementia in primary care: a systematic review

**DOI:** 10.1186/1471-2296-11-52

**Published:** 2010-07-01

**Authors:** Tamar Koch, Steve Iliffe

**Affiliations:** 1 Department of Primary Care & Population Health, University College London, Royal Free & University College Medical School, Rowland Hill Street, London, NW3 2PF, UK

## Abstract

**Background:**

The diagnosis of dementia in primary care is perceived as a problem across countries and systems, resulting in delayed recognition and adverse outcomes for patients and their carers. Improving its early detection is an area identified for development in the English National Dementia Strategy 2009; there are thought to be multiple benefits to the patient, family, and resources by doing this. The aim of this review was to carry out a rapid appraisal in order to inform the implementation of this policy.

**Method:**

Publications in English up to August 2009 relating to barriers to the recognition of dementia, were identified by a broad search strategy, using electronic databases MEDLINE, EMBASE, and psycINFO. Exclusion criteria included non-English language, studies about pharmacological interventions or screening instruments, and settings without primary care.

**Results:**

Eleven empirical studies were found: 3 quantitative, 6 qualitative, and 2 with mixed methodologies. The main themes from the qualitative studies were found to be lack of support, time constraints, financial constraints, stigma, diagnostic uncertainty, and disclosing the diagnosis. Quantitative studies yielded diverse results about knowledge, service support, time constraints, and confidence. The factors identified in qualitative and quantitative studies were grouped into 3 categories: patient factors, GP factors and system characteristics.

**Conclusion:**

Much can still be done in the way of service development and provision, GP training and education, and the eradication of stigma attached to dementia, to improve the early detection and management of dementia. Implementation of dementia strategies should include attention to all three categories of barriers. Further research should focus on their interaction, using different methods from studies to date.

## Background

There are approximately 700,000 people living with dementia in the UK today, and the number is set to double by 2040, resulting in current costs of care of £17 billion spiraling to £50 billion each year [1]. Similar demographic shifts in all industrialized countries will produce similar increases in the prevalence of dementia syndrome, with the impact being greatest in lower and middle income countries [2]. Dementia is one of the leading causes of disability for older people. From a Global Burden of Disease perspective it contributes 11.2% of all years lived with disability which is higher than stroke (9.5%), heart disease (5%) and cancer (2.4%) [3]. From a primary care perspective however, dementia may not impact greatly on the workload of an average General Practitioner, who might currently diagnose one or two new patients each year (in a demographically average area) and have 12 to 15 patients with dementia in a list of 2000 [4]. This workload will change as populations age.

This changing demography challenges governments and other health care providers to develop and improve services to people with dementia, with an emphasis on earlier diagnosis, provision of support in the community, and the role of primary care services [5,6]. National plans for the development of dementia services have appeared in France, the Netherlands, Norway, Cyprus, England [7], Australia [8] and New Zealand [9]. The English National Dementia Strategy [1] sees General Practitioners (GPs) as being well placed to recognise the symptoms and signs of early dementia, and to trigger the necessary investigations to establish a diagnosis [9]. However, position does not necessarily lead to action, and there appear to be barriers to recognition of dementia in primary care.

There are concerns about diagnosing dementia because of potential negative impacts on the patient (e.g. stigma, missing a different and treatable diagnosis like depression), the caregiver (longer time in a stressful role), and service provision (overloading of specialist services, diagnostic uncertainty, difficulty in changing provision) [10]. In contrast, there are thought to be a wide range of benefits to the patient, the family and caregivers, and to resources and services, in diagnosing dementia earlier, and it has been suggested that these far outweigh the risks. For example, timely diagnosis enables the early initiation of treatment (including pharmacological treatment), and it has been shown that this can delay admission to nursing homes and time to dependency [11,12]. In addition, earlier diagnosis can prolong the early phase of the condition, thus shortening the moderate to severe stage, and its associated burdens [13]. Earlier detection would also allow the opportunity to plan for the future, and promote awareness of relevant support agencies and organisations [14]. This can also help relieve the psychological distress experienced by caregivers [15].

Despite this, early detection of dementia is difficult to achieve in primary care across different health care systems [16-18] with diagnosis taking between 18 to 30 months, and in the extreme, up to 4 years [19]. There have been a number of reviews of the reasons for this difficulty, and the barriers involved in diagnosing dementia in a timely manner. Firstly, detection could be delayed for reasons attributable to the patient, the patient's family, or caregiver. The symptoms of dementia may not be recognised by the patient or family, and some of the early characteristics such as memory loss, functional disability, or emotional lability might be thought to be a 'normal' component of ageing [20]. Dementia in itself results in lack of insight and so many patients are unaware that they have a problem [21]. This could delay diagnosis in isolated patients who live alone, and often it is the caregivers who make an initial diagnosis [18]. In addition, the stigma that can be attached to the condition may prevent the patient or family from seeking medical help, because of embarrassment, shame, or uncertainty [22].

Primary care practitioners may also be responsible for a delay in reaching a diagnosis for a variety of reasons such as knowledge gaps [14], the lack of a definitive diagnostic test leading to diagnostic uncertainty and concerns of mis-labelling patients [10,22], and stigma within the medical profession. This hypothesis is supported by Eefsting et al's study [23] which showed that when a label of 'cognitive impairment' was used, the sensitivity of diagnosis by GPs improved. Other reasons that might influence GPs are the amount of time necessary to invest in what is often a hypothetico-deductive and longitudinal process of diagnosis [14], the (lack of) financial reimbursement, and the perception that making the diagnosis produces little practical benefit for the patient [24].

Specialist services for the management of patients with dementia have been varied in the UK. This has led to uncertainty on the part of the GPs about access to resources, which may have contributed to a variable quality of management delivered to the patient. It is only with the growth in interest in dementia services that an effort has been made to ensure that services are uniform, that adequate training and development are provided for health care professionals, and that regular monitoring and evaluation of service provision takes place [1].

The aim of this review is to systematically investigate current evidence about the barriers to dementia diagnosis in primary care, in order to address and overcome them. Because the implementation of the National Dementia Strategy is already underway in England we carried out a rapid appraisal of the literature to help inform strategy implementation at an early stage. Because the English National Dementia Strategy is only one of a raft of policy initiatives across the world we believe our findings may be relevant to other countries.

## Method

### Search strategy

A rapid appraisal approach was adopted to inform the current implementation of policy. Health care policy is influenced by many factors other than research evidence, including political pressure, ideological stance and the need to take action. Influencing policy development and implementation is now an issue for researchers in all those countries with government-driven dementia strategies, and as Black has pointed out [25], timing is important: "Windows of opportunity to make change open up only rarely and briefly, when policy makers' values happen to coincide with the implications of research".

A systematic search for articles in the English language pertaining to the detection of dementia in the community was made using the electronic databases MEDLINE, EMBASE, and psycINFO, without restricting the date or language of publication. Searches were carried out between May and August 2009. A broad research strategy was adopted using the terms listed:

Dementia   OR Cognitiv* Impair*   OR Alzheimer's Disease

AND

Primary Care   OR General Practi*   OR Family Pract*

AND

Diagnos*   OR Manag*

AND

Educat*

A two-step process was carried out in the electronic search, with educat* added as the second step, after all the other search terms. The terms used were text words that were searched for in the abstracts. Bibliographies of articles discovered were examined for additional relevant literature.

### Search selection

This search resulted in a total of 4311 articles being identified in the various databases, although many of these were duplicates. To prevent narrowing of the search scope and therefore potentially reducing the search sensitivity, the terms were not refined, but instead each title was reviewed - with its abstract if available - in order to ascertain its relevance. A number of exclusion criteria were applied. All studies about pharmacological interventions (for dementia or Alzheimer's Disease) were excluded, as were studies relating to the validity or usefulness of specific cognitive function tests. If the searches identified articles about the diagnosis or treatment of dementia in anywhere but a primary care setting (for example, the benefits of respite in long-term care facilities), if the article related to a cohort that was not relevant to this review (for example the evidence for various interventions for care-giver health), or if the paper was merely a clinical discussion about dementia diagnoses or care, then it was excluded. Letters were also excluded, as were publications in languages other than English.

After applying these criteria, 160 articles remained, but seventy of these were unavailable or unobtainable online and these were also excluded for pragmatic reasons. A main objective of the review was to inform the implementation of the National Dementia Strategy [1], which is already underway. Because it would take a significant amount of time to locate and access the articles that were not readily available, which would delay the dissemination of the results of the review, we opted to exclude the seventy. Moreover, this review - rather than systematically aggregating data - has adopted a more interpretive approach [26], the purpose of which is to construct theories grounded in the research, develop an understanding about the origins of the barriers, and to generate practical methods to overcome the barriers, through changing practice and through well-designed future research.

### Data Extraction

**Figure 1:**
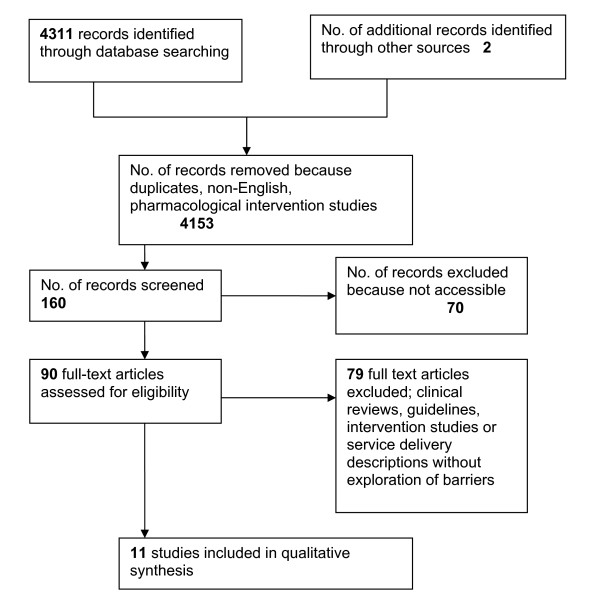
**PRISMA flowchart for Barriers systematic review**.

Each of the remaining articles were scrutinised by the two authors (SI & TK), and subsequently categorised into one of three topics. These were: 1) barriers to diagnosis and/or care, 2) studies of or guidelines about diagnosis and management, and 3) studies of changing provision or practice in dementia care in the community. Figure 1 shows the search process, presented according to PRISMA guidelines [27].

Discrepant categorisations were discussed between the two authors and agreements reached as to which category the paper fell into. For the purposes of this review, studies of barriers to diagnosis or care were examined and data tabulated, to enable the comparison of evidence. The elements examined for comparison included: location and type of study, number of participants, recruitment process, methodology, data analysis, results, and conclusions. Significant references found in relevant articles were explored and data extracted accordingly.

## Results

Eleven primary research studies were identified. Of these, six were qualitative, three were purely quantitative and two used mixed methodologies. Because Primary Care Physicians (PCPs) have different titles (General Practitioners [GPs], Family Physicians [FPs]) in different countries, for the purposes of this article we refer to all doctors in this category as PCPs, unless there is a specific reason for identifying a country-specific label.

Five out of the eight qualitative or mixed studies involved interviews or focus groups with PCPs. The remaining three also included other health care professionals who worked with dementia patients (for example specialist doctors, community nurses), or patients or carers. The quantitative studies about barriers to care all involved questionnaires completed by PCPs, with sample sizes ranging from 116 (35% response rate) [28] to 1005 (55% response rate) [29]. The response rates of the studies ranged from 28% (n = 127) [30] to 67% (n = 153) [16].

Six themes emerged powerfully from the research: Lack of support, time and financial constraints, stigma, diagnostic uncertainty and disclosure of the diagnosis.

### 1. Lack of Support for patient, caregiver, or PCP

All studies identified lack of support, in one capacity or other, as being a major barrier to the detection or management of dementia. This lack of support could take many forms:

• Concerns about the dearth of caregiver support and education [27]

• Insufficient resources for the PCP to provide as much support as sometimes may be expected of them [28]

• Lack of support for the PCP with limited access to secondary services [29,30]. Olafsdottir et al [16] demonstrated that only 43% of PCPs were satisfied with the specialist input that their patients received (which was similar to the Audit Commission's [29] finding of 50.2%).

• Lack of knowledge about or access to community services and resources [31,32]. In Turner et al's study [30], only 45% of the PCPs knew about patient-support services, and 53% knew details of the local memory clinic. 56% of them felt that organizing the necessary social support services was the most difficult aspect of care [16].

• The absence of interdisciplinary teams to enhance management [28,31], with 43% of PCPs feeling care could be improved with the addition of a dementia-trained care coordinator in the primary care centre [16].

### 2. Time Constraints

The PCPs felt that they did not have time ring-fenced in their normal schedule to carry out the necessary procedures, tests and reviews that are needed in order to make a diagnosis of dementia [32,28,31], and this was corroborated by the quantitative studies (83% of those asked in Turner et al [30], 69% in Olafsdottir et al [16]).

### 3. Financial Constraints

Inadequate financial remuneration (especially in countries where PCPs are paid according to services they provide) was proposed by PCPs as a barrier in the qualitative research [33,28,31,34], and was confirmed in one quantitative study (46% of PCPs in Turner et al [30]).

### 4. Stigma

Several studies mentioned concerns about stigma, attributed by the patient or caregiver on receiving a diagnosis of dementia [35]. This may develop as a result of a number of fears, from being labelled as having a mental illness, to loss of independence or autonomy, or having to go into a nursing home [33]. Some PCPs themselves seemed to attach stigma to the condition of dementia by assuming that the patient/caregiver did not want a diagnosis until such time that the symptoms were so severe that it was inevitable [34].

### 5. Diagnostic Uncertainty

Many of the PCPs interviewed expressed concern about uncertainties around making the diagnosis, arising from several perspectives. Some felt that their training for this either as under- or post-graduates had been inadequate and did not feel confident enough to make the diagnosis [34,36], whilst others felt that because at early stages the manifestation of the condition was slow and fluctuating and often overlapped by co-morbidities, or the boundary between cognitive impairment and 'normal ageing' can be blurred, it made the diagnosis particularly complicated to make accurately [32,33,28]. Quantitatively, PCPs in two studies felt they did not have sufficient knowledge of or training about the condition (80% in Olafsdottir et al [16], and 52.4% in the English Audit Commission report [29]), and a minority of PCPs routinely used validated instruments to aid diagnosis (47% in Allen et al [28], 44% in the English Audit Commission report [29]). Olafsdottir et al [16] showed that 39% of the PCPs questioned thought that the early detection of dementia was the most difficult aspect of the condition, and Allen et al [28] showed that diagnosing the cause of dementia was considered to be the highest learning need for the PCPs. On the other hand, 64% of the PCPs in Turner et al's study [30] felt confident in their own ability to make a diagnosis (although 33% of them later went on to support the notion that specialists ought to be the ones doing this), compared with only 32% who felt confident in advising on symptom management. Several conveyed a sense of trepidation about making a false diagnosis or getting it wrong, and the effect that this might have on the doctor-patient relationship [37], or the potential to invoke medico-legal action [33].

### 6. Disclosing the Diagnosis

Most studies revealed that PCPs experienced difficulties in disclosing the diagnosis to the patient or family, for a variety of reasons. In Iliffe & Wilcock's focus groups [36], PCPs acknowledged the risks in disclosing a diagnosis at the wrong place or the wrong time, but also recognised that sometimes the single act of investigating the patient for dementia, in itself can enable patients or their families to understand the possibilities about diagnosis, and may therefore result in some relief. In Teel's study [35], PCPs disclosed that often patients or their families will deny the diagnosis when given to them, often just wanting themselves/family member "fixed". PCPs might get around this by using euphemistic terms, or reframing the situation so that priority is given to providing care and support to the patient rather than a medical label [35,37]. In addition, PCPs conveyed fear that disclosure could again damage the doctor-patient relationship [33]. In Allen's focus groups [28], family practitioners suggested a solution to these issues by proposing a "breaking bad news" approach to disclosure, but allowing confirmation of the diagnosis to be made by the specialist. Barriers to disclosing dementia were also noted in Allen et al's [28] Canadian research. Whilst 72% of the PCPs routinely informed family members of the diagnosis, fewer (31%) informed the patient. The PCP participants in this study also highlighted approaches to disclosure of the diagnosis as being their second highest priority learning need. In contrast, the most frequently identified perceived barrier to care was the lack of insurance reimbursement for anti-dementia drugs.

Two other important themes which emerged, but with less force than the ones above; were delayed presentation and therapeutic nihilism,

### Delayed Presentation

There was a diversity of reasons given for delayed presentation of the patient to primary care services. These ranged from:

• The patient or family being in denial about there being anything wrong [32]

• Families being so immersed in compensating for the loss of function of the patient that they don't really notice that there is something wrong [35]

• Patients or families not having the knowledge to realise it is part of a medical condition, or attributing symptoms to "normal ageing" [35,33].

Cahill et al [34] specifically asked the PCPs whom they blamed for delayed diagnoses, and 35% of the PCPs blamed themselves, with 20% others blaming the family and 11% blaming the patient. Several of the studies identified specific characteristics about the PCPs themselves, their services, or the wider health services that could contribute to the delayed diagnoses. One of the main areas that arose as potentially acting as a barrier to early diagnosis and management was limited knowledge about dementia.

### Therapeutic Nihilism

This describes the attitude on the part of the PCP that diagnosis is pointless or not worthwhile because there are no available treatments or benefits to making that diagnosis, and so it could in theory cause more harm than good. Two studies found that this could contribute to delay in timely diagnosis [34,37]. The English Audit Commission [29] also found that only 52% of PCPs felt that it was beneficial to make an early diagnosis. It was also hypothesised that this attitude could hinder PCPs' awareness of local resources and support services, or even referrals to specialists, in an attempt to protect the patient or family from intrusive or frightening tests [37]. In addition, Turner et al [30] found that female PCPs were less likely to express 'heartsink' attitudes, and that the more experienced PCPs were also more pessimistic.

## Discussion

### Summary of Findings

In total eleven papers with empirical data were found, including six qualitative, three quantitative, and two with mixed methodologies. The six themes from the qualitative studies and the findings from the quantitative studies can be grouped into doctor factors, patient or societal factors, and system factors. The former consists of barriers such as diagnostic uncertainty or insufficient knowledge or experience, as well as disclosing the diagnosis, stigma attached to dementia, and therapeutic nihilism. Patient or societal factors also include stigma, as well as delayed presentation which could be because of stigma, but also because of many other reasons. Finally the systems (in the countries studied) were responsible for many of the barriers discovered, including for example, time constraints and lack of support (which were the most often-identified factors), as well as financial or remuneration issues.

### Strengths & Limitations

This systematic review aimed to investigate what was known about the barriers to the detection and management of dementia, on a background of an established understanding that PCPs do not achieve high rates of early detection, and often express difficulties in managing or following guidelines for dementia patients.

Not all of these studies made explicit the fact that they were investigating barriers in primary care, and so it was a strength of the method that abstracts were read through and articles categorised according to the subject they covered, in order to limit the omission of relevant data. Moreover, none of the UK studies were conducted after the introduction of the dementia domain to the Quality and Outcomes Framework, which - because of the financial remuneration attached to maintaining a register of dementia patients and offering them an annual review - may influence the way PCPs here approach dementia.

Whilst the search terms used were thought to be broad enough to capture most relevant publications, it is possible that there is research that was not caught in our searches, because of the omission of one or other of the strata of terminologies, for example, *primary care *or general practi* or family practi*. There was an attempt to find any missing publications by searching bibliographies from identified articles, but it is possible that not all relevant studies were found. In addition, many of the potentially relevant articles harvested by the search terms were either in a non-English language, or were not readily located or available, so possibly some important or pertinent research which could have enriched this review has been missed. However, only a small proportion of studies were found through searching reference lists, which suggests to us that the literature that we did identify reflects the current state of our knowledge.

The research was carried out in a variety of countries all over Europe and North America which could increase the significance of the common themes identified. Conversely, because the PCPs take on different roles and the health systems are different in each country, it could make it difficult to extrapolate conclusions about the contribution of those factors to the issues of barriers (and therefore make the results less generalisable to the UK), and make future research more difficult to define generally.

All the reviewed studies had some methodological weakness. The sample sizes for most studies, including the quantitative studies, were small on the whole. This is acceptable in qualitative research, but three of the five quantitative or mixed studies had fewer than 200 participants. Furthermore, the recruitment process of some of the studies lacked robustness. With the qualitative research, there were attempts to capture the widest breadth of views with purposive sampling, but some of the studies resorted to random, snow-balling, or convenient sampling techniques, which would not necessarily achieve this. Similarly, whilst some of the quantitative data collection attempts were to recruit generally and randomly, at times there was potential for selection bias in the way the researchers identified their sample from which to recruit.

### Implications for the Future

#### Addressing Patient Barriers

Results from the studies have shown that patients and families are sometimes responsible, at least partially, for a delay in diagnosis. The possible reasons for this have been described earlier, but tend to be due to either the stigma that is sometimes attached to dementia, or lack of knowledge on the part of the patients/families. One strategy to eliminate stigma and enhance attendance is by educating society. There is an overall low level of understanding of the condition of dementia in the public and non-specialist domain which, alongside the stigma that might be attached, makes it difficult for people to talk about [1]. There is also uncertainty about the boundary between normal ageing and cognitive impairment. The English National Dementia Strategy (NDS) [1] devotes an entire chapter to "Raising Awareness and Understanding" (Chapter 3) where it proposes a variety of public information campaigns to deliver education about dementia. The need for this has been corroborated by several other studies [38,39], and the English Alzheimer's Society carried out a pilot awareness campaign in 2007, which achieved positive results [40].

#### Addressing Doctor Barriers

In spite of policies such as the NDS encouraging the early detection of dementia, if the PCPs have not been persuaded of its validity then they may well not adopt any attempts to change. Whilst results have demonstrated the uncertainties that PCPs' face about making a diagnosis, the benefits (as described earlier) are numerous and persuasive and so methods to tackle this hesitancy ought to be explored. Re-framing the condition may help patients, family, and practitioners in dealing with the consequences of such a serious position. Iliffe et al [33] offer the suggestion of re-categorising dementia to a slowly-progressing disability, rather than a condition or disease, thus making it more acceptable. Alternatively, rather than re-framing the condition, de Lepeleire & Heyrman [41] instead propose re-framing the clinical approach to the patient, by considering them as a 'frail elderly', thus enabling an assessment of function alongside the environment. This argument goes on to suggest that maintaining a 'frail balance' with medical, social and psychological support will be the motivating drive for clinicians who might otherwise adopt a stance of therapeutic nihilism. Another subtle yet potentially effective adjustment would be to modify the terminology used from 'diagnosis' to 'recognition' [33]. This would convey a dynamic process, which is continuing rather than finite, may rely on multiple assessments, and would offer more than just a label; an opportunity to manoeuvre the patient and family into a position of acceptance of their situation as well as being receptive to help and support that is available to them.

Results suggest that important areas for future research and development ought to be effective educational and support measures for PCPs. Because the incidence of dementia means that, for example, on average each British GP will encounter only 1-2 newly diagnosed (or potential to be diagnosed with) dementia patients annually, learning from experience cannot be relied upon as an effective educational tool. Instead, educational strategies need to be targeted to PCPs in a way that will effect a change in their clinical practice. Much of the material generated by the PCPs revealed a concern about their own abilities to make a diagnosis of, or manage a patient with, dementia. This was corroborated quantitatively, but even then the data was reflective of PCPs' *perceptions *of their knowledge, rather than their *actual *knowledge or training. Because there is no one particular screening instrument that is recommended for use, because there is no definitive diagnostic test, and because dementia is more of a syndrome than a disease, it is easy to imagine how uncertainty amongst clinicians who do not deal with it regularly might arise. This, dovetailed with the inherent stigma and apprehension about making a wrong diagnosis and disclosing the diagnosis with aptitude and empathy, seems to be the font of insecurities that make PCPs reluctant or unable at times to recognise dementia earlier. So, rather than simply augmenting clinical knowledge, educational interventions should ideally be more attitudinal, focusing on enhancing PCPs' perceptions of their suitability and ability to make the diagnosis, and the value of doing so in a timely manner.

#### Addressing System Barriers

Much of the difficulty in detecting and managing dementia for PCPs arises from the health systems in which they work, rather than their own abilities, and this was a sentiment that was expressed by PCPs from Europe as well as North America. In many cases, part of the problem was due to the way the primary care system was organised or arranged, for example, with timings and finances. However, most of the systemic obstacles stemmed from a feeling of lack of support from and communication with secondary specialist care. In addition, it has been suggested that diagnosis would be best-achieved by a two-step process, where the PCP detects cognitive impairment and loss of function and suspects dementia, but the actual diagnosis and sub-typing is confirmed by a specialist [42]; this could lead to confusion, and is dependent upon good communication and access between the generalist and specialist. To some extent, services provided will be dependent on local provisions, demography, and geography, but even if the services are available, if the relationship or understanding of expectations between the generalists and specialists is dysfunctional, then patients will not receive the high quality of care that they deserve [33]. Therefore, there is potential to conduct research into what support PCPs envisage as being helpful, their perceptions and expectations around their relationship with secondary care, and what they feel they would need in order to feel confident about detecting and managing dementia. Shared care protocols would be one technique that could be used to attempt to divide care appropriately between the generalist and the specialist, but which could provide the PCP with the knowledge and support that they need. This has been substantiated by Renshaw et al [43] who discovered that if the local secondary services had made contact with and gave education about dementia to their local PCPs, the PCPs were significantly more likely to view early diagnosis as beneficial compared to those that hadn't. Presumably, through the process of development of local protocols the roles of primary and secondary care in diagnosis and management will be discussed and defined, providing much-needed clarity for all potential stakeholders [44]. In addition it has been suggested that referral pathways are more effective than simple guidelines, and that a clear and robust multi-disciplinary infrastructure will enable diagnosis and management [45], and enhance patient and caregiver satisfaction [31]. Research can be carried out in order to establish effective educational tools which will reinforce the advantages of shared care, and the use of multi-disciplinary teamwork for management.

## Conclusion

Understanding the factors that can promote diagnostic delay could inform intervention studies, which we will investigate further in a separate review. However, the ways in which the main factors interact may determine the timeliness of diagnosis, so further research could usefully address this, perhaps using significant event analysis methods in cases of late presentation and very early diagnosis.

## Competing interests

SI was a member of the External Reference Group of the National Dementia Strategy, has received unrestricted funding for educational research from the pharmaceutical industry, and has acted as an advisor for industry - supported educational programmes in Europe

## Authors' contributions

TK and SI decided on the research strategy, and TK carried out the search and filtered out publications not fitting the inclusion criteria. Both authors reviewed papers and agreed on their classification, and both have contributed to the writing of this paper.

## Authors' Information

Tamar Koch is a Clinical Associate of Academic Primary Care in the Department of Primary Care and Population Health, at University College London.

Steve Iliffe is Professor of Primary Care for Older People at University College London and chief investigator on the EVIDEM (Evidence-based Interventions in Dementia) programme http://www.evidem.org.uk. The EVIDEM programme receives financial support from the National Institute for Health Research (NIHR) Programme Grants for Applied Research funding scheme. The views and opinions expressed in this editorial are those of the authors and do not necessarily reflect those of the NHS, NIHR or the Department of Health

## Pre-publication history

The pre-publication history for this paper can be accessed here:

http://www.biomedcentral.com/1471-2296/11/52/prepub
